# EateryTag: investigating unobtrusive edible tags using digital food fabrication

**DOI:** 10.3389/fnut.2025.1641849

**Published:** 2025-10-15

**Authors:** Yamato Miyatake, Parinya Punpongsanon

**Affiliations:** Graduate School of Science and Engineering, Saitama University, Saitama, Japan

**Keywords:** edible tag, human-food interaction, 3D food printing, digital gastronomy, unobtrusive tagging, computer vision

## Abstract

Human-Food Interaction (HFI) examines how digital integration can enhance dining experiences, with food tagging playing a crucial role in connecting physical dining environments with digital information. Attaching optical tags to the surface or sides of food often detracts from its aesthetics, negatively impacting the perceived taste and overall dining experience. To address this issue, we propose an unobtrusive food tagging approach that embeds tags inside the food, maintaining both its visual appeal and sensory qualities. We first developed a tagging method using a 3D printer and proposed an end-to-end pipeline for embedding and retrieving the tags. We evaluated this method in terms of tag detectability, concealability, and the eating experience. Additionally, we developed tagging methods using molding and stamping to extend acceptability to the traditional cooking environment. Through a workshop with three home chefs, we found that these methods are accessible and easy to adopt for novice users. Our findings demonstrate the potential of embedded food tagging to integrate digital information into the dining experience without compromising culinary integrity. This approach offers new directions for HFI research and practical applications.

## 1 Introduction

Human-Food Interaction (HFI) is an emerging interdisciplinary field that harnesses advanced digital technologies to enhance food-related experiences (1). A growing focus within HFI is personalized food, in which recipes, textures, and appearance are tailored to individual preferences, health conditions, or cultural contexts (2). Recent advances in digital fabrication [e.g., 3D food printing (3–5) and computationally-designed molding and stamping (6–8)] enable precise control over the geometry, internal structure, and sensory attributes of food to realize such personalization. Unlike mass-produced food, each personalized item is associated with unique metadata describing its intended consumer, allergy information, and nutritional profile. Preserving this metadata with the food itself is crucial for enabling traceability, assisting preparation, and enhancing user experiences.

Existing approaches to embedding data into food fall into three broad categories. The first approach is to attach machine-readable tags to packaging, such as barcodes or QR codes (9), which offer high data capacity but are discarded with the package. The second is creating edible markers using surface modifications (10–14), which are food-safe but can be visually intrusive and may alter the eating experience. The third is leveraging intrinsic material properties (e.g., electrical conductivity) to encode information without altering appearance (15–17), though these methods are constrained by lower capacity and limited compatible materials. These limitations underscore the need for methods that can integrate rich, interactive data into food without compromising safety, appearance, or sensory quality.

We propose EateryTag, a method for embedding edible tags inside food that can be read by illuminating it from the backside. EateryTag encodes data by forming two distinct internal geometries with different light absorption ratios. When directional light passes through the food, the contrast between these geometries reveals the embedded pattern on the surface, making it machine-readable, while remaining invisible to diners under ambient lighting (e.g., ceiling lights). Unlike prior methods, EateryTag preserves the foods appearance and keeps data intact until consumption by hiding tags within the edible portion. It also supports conventional fiducial markers such as QR codes and ArUco markers, enabling robust and efficient decoding without relying on specific material properties. We were inspired by previous work in additive manufacturing with non-edible materials such as plastics that embed unobtrusive tags using internal structures (18–20), and we adapt this concept to the food domain to enable safe, visually hidden, and machine-readable tags inside edible items.

**Figure 1:**
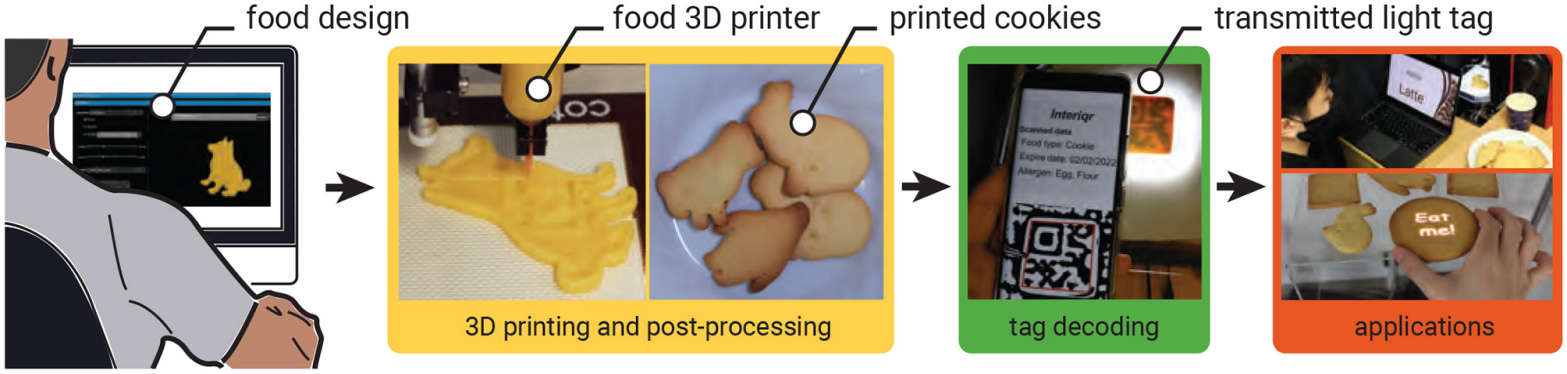
*EateryTag* is a method that utilizes the infill structure of the 3D printing process to embed information inside the food, which allows for hiding the tag from the human eye. We present an end-to-end pipeline that allows users to embed data through 3D food printing and decode it through several applications.

To realize EateryTag, we developed three fabrication workflows: 3D food printing, molding, and stamping. 3D food printing offers precise, automatic, end-to-end control over geometry, allowing seamless integration of tags during fabrication (Figure 1). Our interface determines tag content and geometry from user inputs such as encoded information and desired size, and applies one of two embedding strategies using either air gaps or colored dough. (detailed in Section 2.4). We evaluated the tags embedded through this process in terms of readability, concealability, and eating experience to validate the effectiveness of the 3D printing method.

The molding and stamping methods are designed to extend material compatibility and simplify integration into conventional cooking environments where a food 3D printer is unavailable. In the molding method, we create the tag shape by filling a cell-shaped mold with base and colored food materials, inserting it into the target food, and covering it with an additional layer. In the stamping method, we use an edible ink stamp to imprint the tag onto the food surface, concealing it afterward. Since both techniques rely on standard kitchen tools and manual processes rather than specialized fabrication equipment, they offer a more accessible and practical alternative for embedding edible information. A small workshop with three home chefs demonstrated that non-experts can effectively produce tags using both methods without specialized training.

### 1.1 Extension from previous iterations

We previously presented a preliminary system in earlier work (21, 22) called *interiqr*. In (21), we introduced the core idea of embedding data inside food using infill structures generated by 3D printing. Follow-up work (22) demonstrated several application scenarios for the system.

In this paper, we extend the prior system by introducing two additional fabrication methods, including molding and stamping, for generating edible tags. These new methods are designed to address the general limitations of 3D food printing, such as the need for paste-like ingredients with tightly controlled material properties (e.g., viscosity and particle size) as discussed in (21). We show that molding and stamping support a broader range of food materials and can be applied in diverse cooking environments. To assess their practical applicability, we conducted a workshop to evaluate how well these methods integrate into conventional kitchen settings.

## 2 3D food printing method

**Figure 2:**
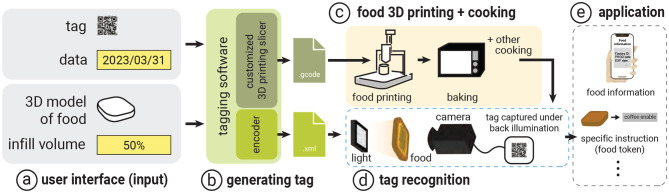
System workflow: **(a)** The system takes the tag information, food 3D model, and infill information as input. Then, **(b)** the system generates a tag by customizing the 3D printing slicer and output G-code file to **(c)** the food 3D printer. At the same time, the system generates an encoder file for **(d)** the recognition process. The 3D printed food is recognized through image processing, and **(e)** the food information is extracted along with other specific applications.

Our main contribution in the 3D printing method is a framework that allows the user to *embed* data into 3D-printed food and later *decode* it for their personal use through its food infills (Figure 2).

The 3D printing method *embeds* tags inside food by intentionally calculating the amount of infill and generating infill structures while keeping the foods original shape and volume. For instance, if users intend to print food with 100% infill, the system generates the tag by switching between different materials with the same taste as the infill (see Section 2.4.2). Otherwise, if the food contains infill spaces (e.g., the user wants less food while maintaining the same appearance), the system generates the tag by designing the infill structure, which determines the printing patterns and produces the control file (i.e., G-code) for the printing extruder to follow (see Section 2.4.1).

Our system then *recognizes* the embedded data from the food tag fabricated during the printing process. For example, after users take a picture of the food under backlight illumination, the system applies image processing techniques to extract and correlate the features of the internal structure in order to retrieve the data (see Section 2.5).

### 2.1 Choosing a target food

In principle, the 3D printing method is applicable to foods that can be extruded through a syringe nozzle (i.e., foods with specific granularity and viscosity) and can maintain their structure after printing. We evaluate our approach using *cookie dough* as the primary material, as prior studies in 3D food printing (4) and food interaction (11, 25) have also used cookies. Using this common reference allows our results to be compared more directly within the existing food-interaction framework. Cookie dough is also easy to control in both form and structure, even after printing. While demonstrating the 3D printing method with cookie dough, we also conduct preliminary experiments with other food materials to explore the broader applicability of our method (see Section 4).

### 2.2 Workflow for the 3D printing method

The workflow of the EateryTag tagging system consists of a *tagging interface* and a *recognition application*. We describe how we use (1) the tagging interface to assign a unique QR code to each instance of a 3D-printed food item prior to printing, and (2) the recognition application to recognize each food tag. The concept can also be applied to the molding and stamping methods detailed in Section 3.

#### 2.2.1 Tagging interface

**Figure 3:**
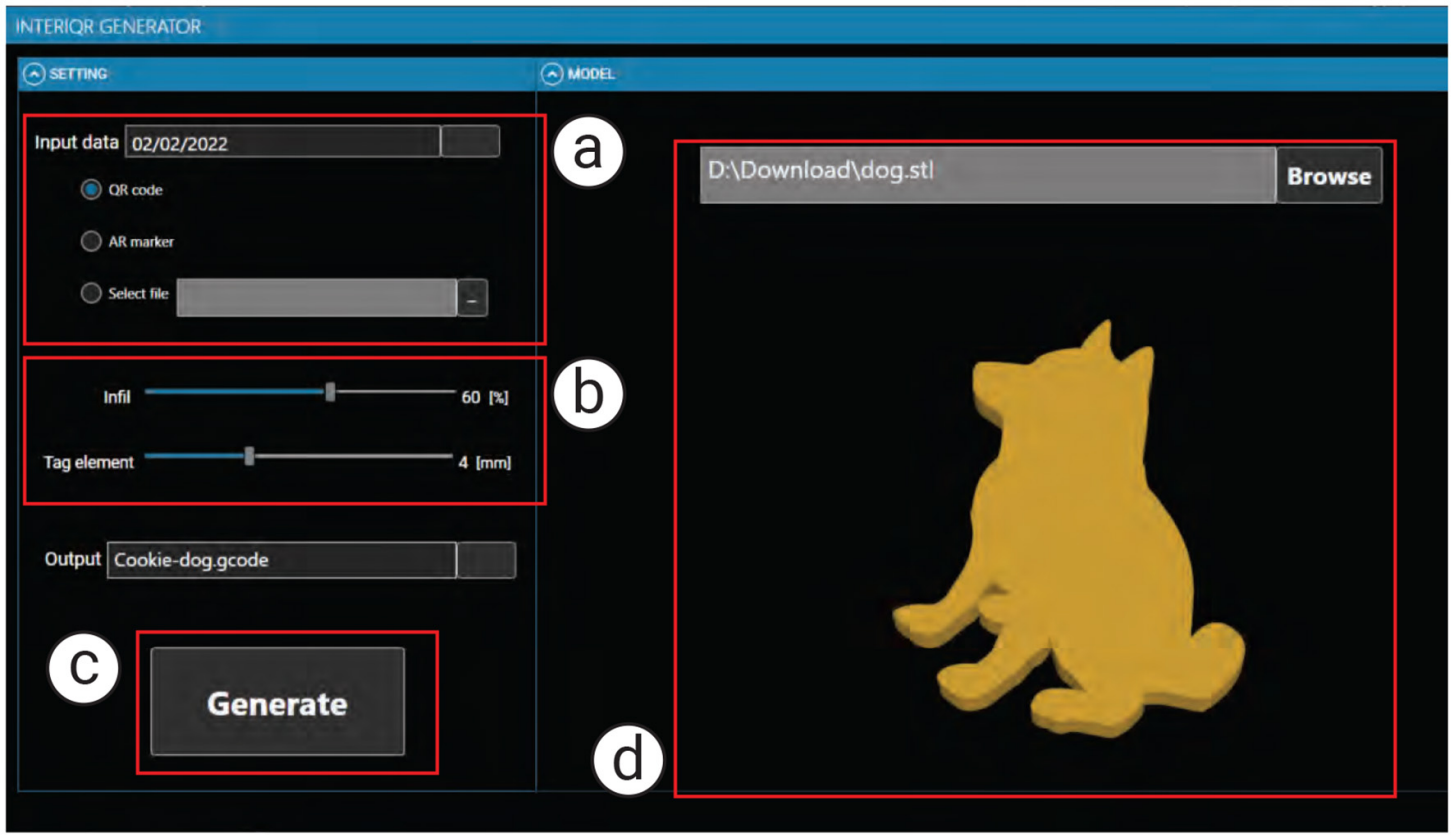
The EateryTag tagging interface takes **(a)** data to be embedded, **(b)** the amount of infill and size of tag (optional), and **(c)** the food 3D model as inputs. Once the user presses the **(c)** generate button, the system embeds a tag inside the food 3D model and exports the G-code to the 3D printer **(d)** shows a preview of the food 3D model with the embedded tag.

As shown in Figure 3, the tagging interface takes as input (a) the data to be embedded and (b) the infill percentage (e.g., from 5% to 100%). In our example, we embed the “expiration date” into a cookie with 60% infill by entering this information into the interface panel.

##### 2.2.1.1 #1 Computing infills and select tagging strategy:

We set the cookie to have 60% infill as the desired volume. Our software calculates a feasible printing path that creates the tag within this infill configuration. Since the infill occupies about half of the total volume, the software leverages the air space and aligns the infill structure using specific slicing parameters.

##### 2.2.1.2 #2 Entering data and generating tag:

We enter the data to be embedded, which can be text or a URL. In our example, we embed the expiration date so that users can check it before eating the cookie.

##### 2.2.1.3 #3 3D printing:

Once all parameters are set, we click the “Generate” button. The software calculates the geometry needed to embed the tag and generates both the control program for the 3D printer (G-code file) and a digital markup file (XML). The XML file stores the tag information after data extraction through the application. Finally, we send the G-code file to the 3D printer to fabricate the tagged food.

### 2.2.2 Tag recognition

Our system provides a stationary setup with backlight illumination and a camera. The user places the food on a plate with the light source positioned behind it. The system automatically captures a top-view image of the food, processes the image, and retrieves the embedded data. Similar to mobile applications, once the image is captured, the embedded data are displayed on the screen. In this case, the expiration date of the cookie is presented to the user.

### 2.3 Material preparation

**Figure 4:**
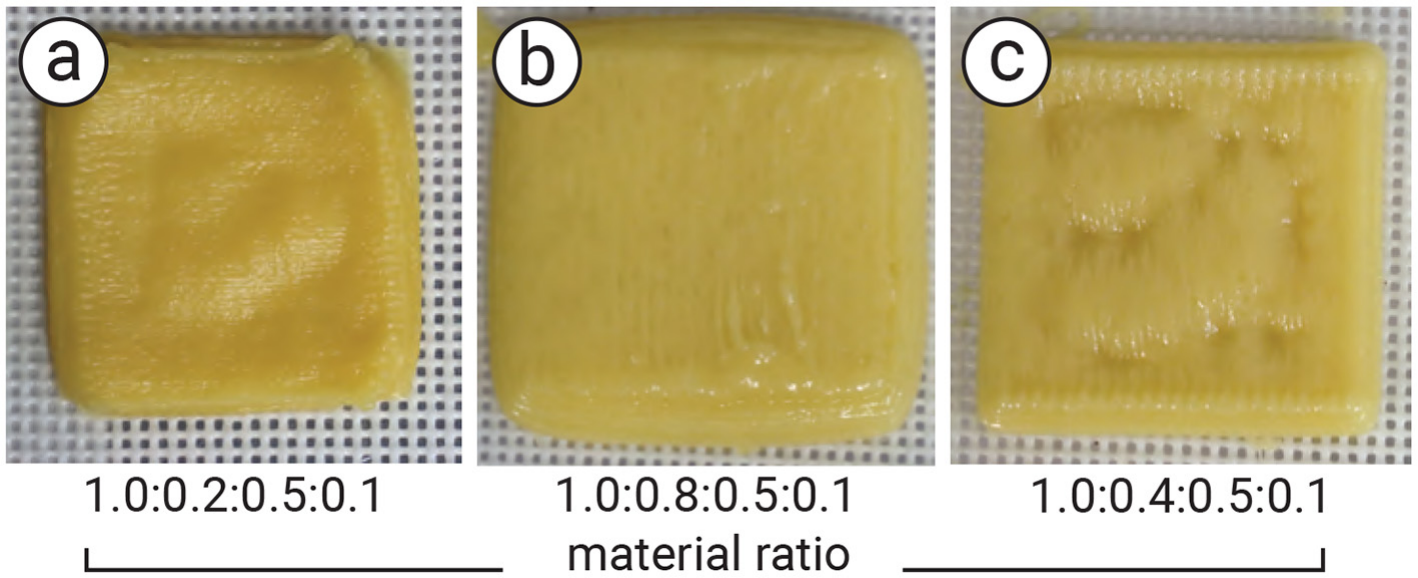
Result of the experiment to examine the blending ration of the cookie dough for 3D printing: **(a, b)** the mismatch ratio of flour, sugar, egg, and shortening; therefore, the printed cookie is too soft and cannot retain the shape, and **(c)** the suitable ratio of cookie dough for our 3D printing. The shape is stable, whereas some part of the surface is collapsed due to the infill structure. The ratio of each cookie dough is shown below the image.

The main challenge in preparing the cookie dough is controlling its viscosity, which directly affects structural fidelity after printing. We conducted a preliminary experiment to determine the optimal blending ratio so that the doughs viscosity is suitable for syringe-based 3D printing. This ensures that the printed cookie closely matches the input 3D model and preserves the intended infill geometry. As shown in Figure 4, we heuristically adjusted the ratios of flour, sugar, egg, and shortening, and found that a 1.0:0.4:0.5:0.1 ratio (Figure 4c) yields the best printability with our syringe-based 3D printer (Nordson EFD Automated Dispensing System). In particular, the structure of the 3D-printed cookie retained its shape, although some surface areas collapsed; possible solutions are discussed in Section 2.6.2. Additionally, we used a small amount of commercially available black food colorant (organic-based) into the dough to produce a blackened material used in the multi-material printing method. Once blended, the dough was rested in the fridge for one hour to set its shape before being filled into the syringe (Nordson Optimum Syringe 20CC) and attached to the printer. The final viscosity of the cookie dough before printing was measured with a viscometer (TGK TVB-10M) to confirm the stability of the blended ratio. The uncolored dough was used not only for the data layer containing the tag, but also for the bottom and top layers surrounding the tag.

### 2.4 Generating tag

Our tagging method uses the infill ratio to determine the tag fabrication strategy: (1) when the infill is less than 70%, we utilize the air space inside the food, and (2) when the infill exceeds 70%, we use a secondary material, such as food with a different color, to embed the tag.

Our software processes the 3D model of the food, similar to a standard 3D printing slicer, to generate the printing paths. It takes the target tag image (e.g., QR code or AR marker) and generates a separate path for it. The tag path is then combined with the top and bottom layers of the original printing path. Finally, the software optimizes the infill structure so that the infill volume matches the specified parameters.

As mentioned in Section 2, we use *cookie dough* as the target food for embedding a tag. The sample tag is a binary tag consisting of 13 × 13 modules (i.e., a micro QR code), which can represent about six alphabetic characters or ten numeric digits (9). The number of modules can be increased depending on the amount of data to be stored.

#### 2.4.1 Utilizing infill and air space

When the infill is less than 70% (i.e., some air space is required inside the food), our method encodes the binary “0” using the infill material and the binary “1” using the air space generated by 3D printing. First, we calculate the amount of infill material needed to produce a standard rectilinear structure, and then determine the material required to generate the tag. For example, if the cookie size is 5 cm × 5 cm × 0.8 cm, printing it with 100% infill requires 10 g of dough. Setting the infill to 70% allocates 2 g of dough for the shells (i.e., the exterior), leaving (10 g − 2 g) × 70% = 5.6 g for the infill. We then adjust the internal structure size to match the target infill amount (e.g., for the fixed tag size) and combine the tag and shell models before generating the G-code for printing.

#### 2.4.2 Using multi-materials

**Figure 5:**
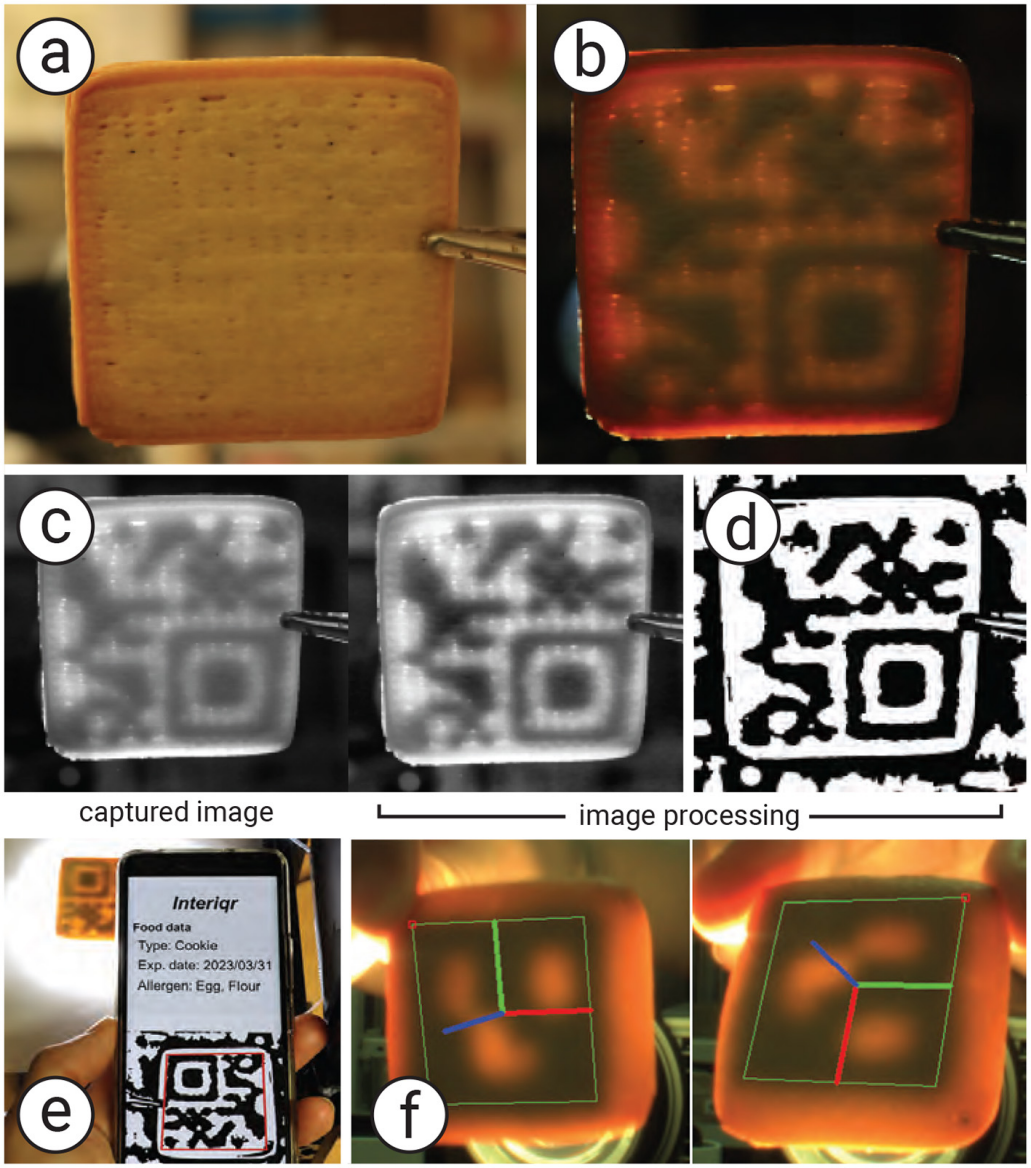
Proposed method to generate and recognize a tag. **(a)** Cookie in a normal view without illumination, **(b)** cookie under backside illumination, **(c**) the image processing process to obtain **(d)** the binary code to recognize the tag using a mobile application. **(e)** The user can use a standard QR reader to recognize the tag and **(f)** obtained the data.

When the target 3D-printed food has an infill greater than 70%, the empty space inside is insufficient for generating the tag. To address this, we print the infill using a secondary material. The material is chosen under specific conditions (see Section 2.6) so that it can be clearly detected by the camera while being enclosed by the standard material. The secondary material represents binary code “1,” while the standard material represents binary code “0.” In our case, we use cookie dough mixed with black food coloring as the secondary material so that it can serve as the tag while retaining the same taste. As a result, the tag remains hidden to users (Figure 5a), even though it is printed with colored material.

### 2.5 Recognizing tag

**Figure 6:**
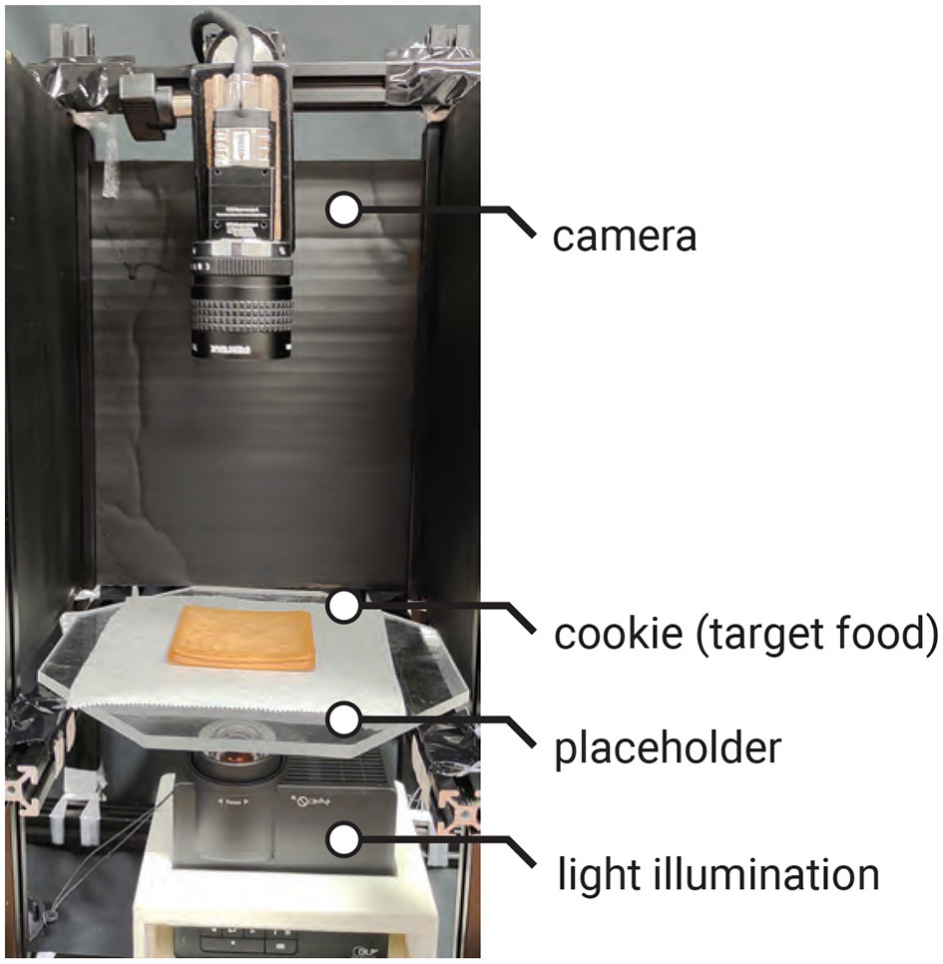
Tag recognition setup: the projector is used to illuminate the cookie from the **bottom**, and the camera captures the image of the cookie from the **top**.

As shown in Figure 6, to recognize the tag, the cookie is placed under a bottom-illumination setup, with a camera (MQ013CG-ON, Ximea) positioned above it. The backlight illumination can be either visible light (e.g., white light), invisible light (e.g., infrared; see Section 2.6.1.1 for details), or spatially coded light. In our sample, we use a projector (PJ WXC1110, RICOH) for backlight illumination, which allows easy control of light intensity and color. We also employ an infrared backlight source (Advanced Illumination Backlight, 880 nm).

The image preprocessing and decoding steps are summarized in Algorithm 1. The captured image is converted to grayscale, processed with adaptive histogram equalization (CLAHE) to increase contrast, and smoothed with a Gaussian blur to reduce noise (Figure 5c). It is then binarized using the adaptive Gaussian threshold method to extract the tag shape (Figure 5d). QR codes are recognized using a standard QR library (e.g., QRQR, DENSO WAVE). In our example, we also use the ArUco library to recognize ArUco markers. Figures 5e, f show, respectively, the decoding process using the QR library and the decoded result using the ArUco library for a 3D position tracking application.

**Algorithm 1:** Tag decoding pipeline.

**Require:** RGB image *I*
**Ensure:** Decoded tag payload, or Failure
1: *G*←TOGRAYSCALE(*I*)
2: *G*_*c*_←CLAHE(*G*)
3: *G*_*s*_←GAUSSIANBLUR(*G*_*c*_)
4: *B*←ADAPTIVETHRESHOLD(*G*_*s*_)
5: *d*_message_←DECODE(*B*, {QR, ArUco})
6: **return** *d*_message_

### 2.6 Experiments

We conducted a series of experiments to evaluate the readability of our edible tags, as well as to verify the feasibility and scalability of our method.

#### 2.6.1 Tag readability

We evaluated tag readability under different backlight illumination conditions and specific light spectra.

##### 2.6.1.1 Transmission spectra

As mentioned in Section 2.5, our system utilizes back illumination to recognize a tag from the captured image. The light is transmitted through the cookie and captured by the camera. Areas that do not block the light, such as air spaces, transmit light across the surface more effectively than areas containing the infill structure, which blocks the light. Moreover, the spectrum of the illumination also affects how light is transmitted through the food material. Selecting the appropriate wavelength band can improve the readability of the tag.

**Figure 7:**
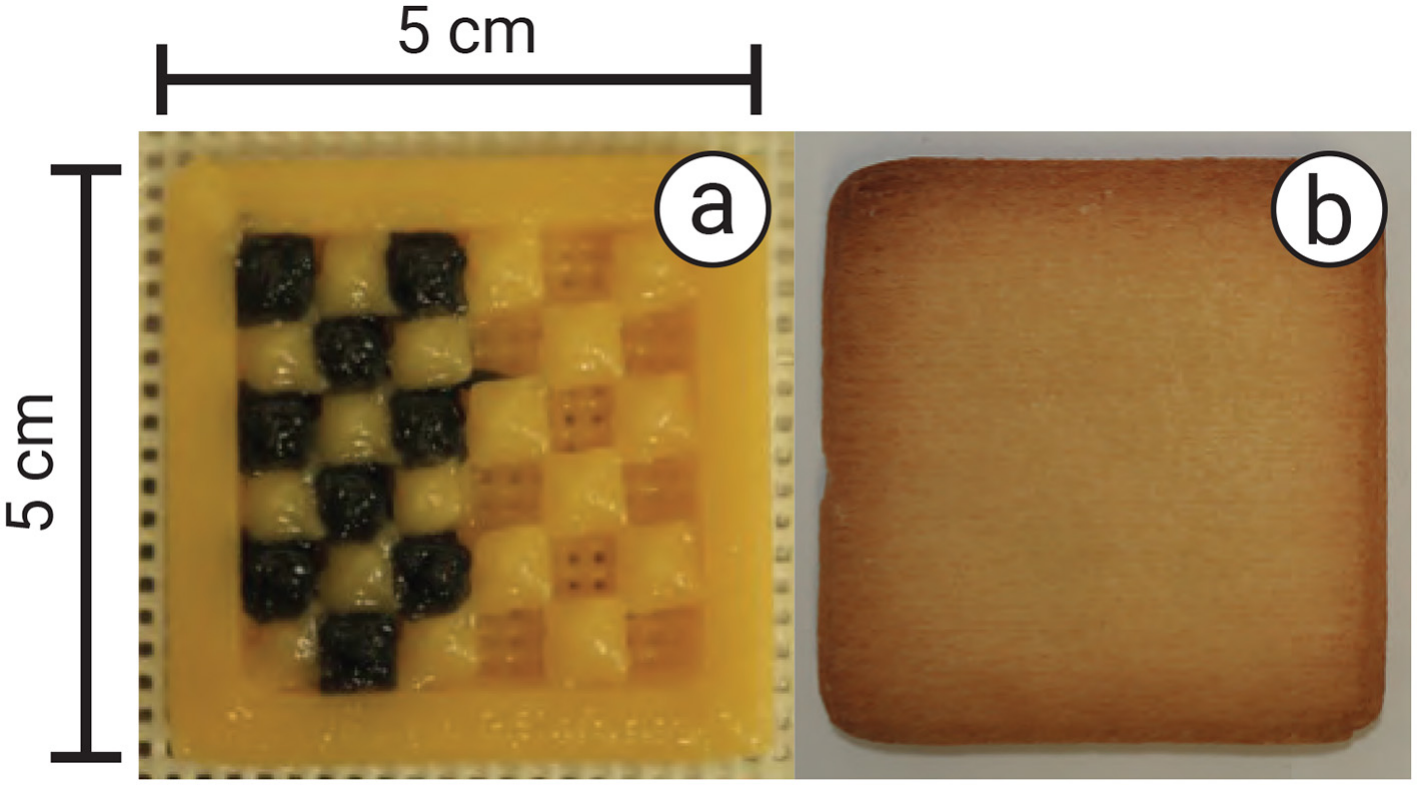
Experimental setup: **(a)** cookie filled with black material and air spacing in a checkered pattern, and **(b)** after being enclosed with layers 1.5 mm in height and baked.

To examine the effect of transmission spectra, we conducted an experiment with various light spectra to investigate the transmission properties of our tags. We used a spectroradiometer (SR LEDW, TOPCON) to measure the transmission spectra of cookies printed with air space, regular material, and black material. In this experiment, we 3D printed a cookie with a checkered pattern, where the infill was filled with black material on the left half and left as air space on the right half (Figures 7a, b). The infill was 1.5 mm in height, enclosed by top and bottom layers of the same height, totaling 2 mm. The light illuminated the backside of the cookie, and the spectroradiometer captured the transmission from the front side.

**Figure 8:**
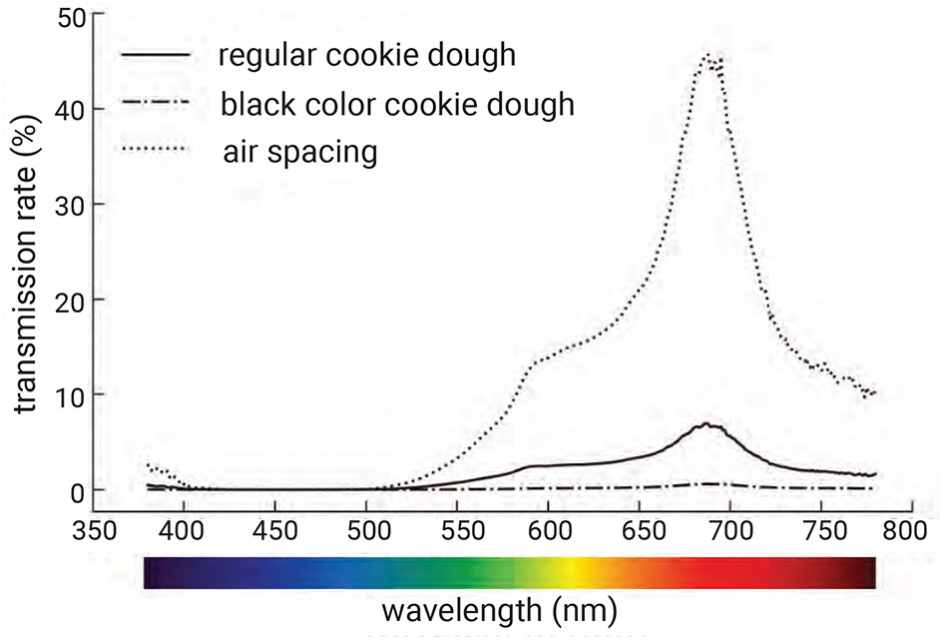
Transmission spectra of different infills: regular cookie dough, black-colored cookie dough, and air space. Black dough significantly reduces transmission compared to regular dough, while air space shows much higher transmission. The contrast is particularly pronounced in the red wavelength region, enabling reliable tag recognition by exploiting this difference.

Figure 8 shows the measured transmission spectra. We found that, with illumination in the wavelength range from 550 nm to 780 nm, the areas containing air spaces appeared brighter than those with black cookie dough or regular cookie dough. In particular, illumination at a wavelength of 680 nm (red light) enhanced the contrast between the tag regions containing air space, black cookie dough, and regular cookie dough. The black-colored dough showed low but nonzero transmission across most wavelengths, although ideally it should be zero since black was used for colorizing, which may be attributed to incomplete light blocking by the colorizer, scattering through the top layer, or minor measurement noise in the optical setup.

**Figure 9:**
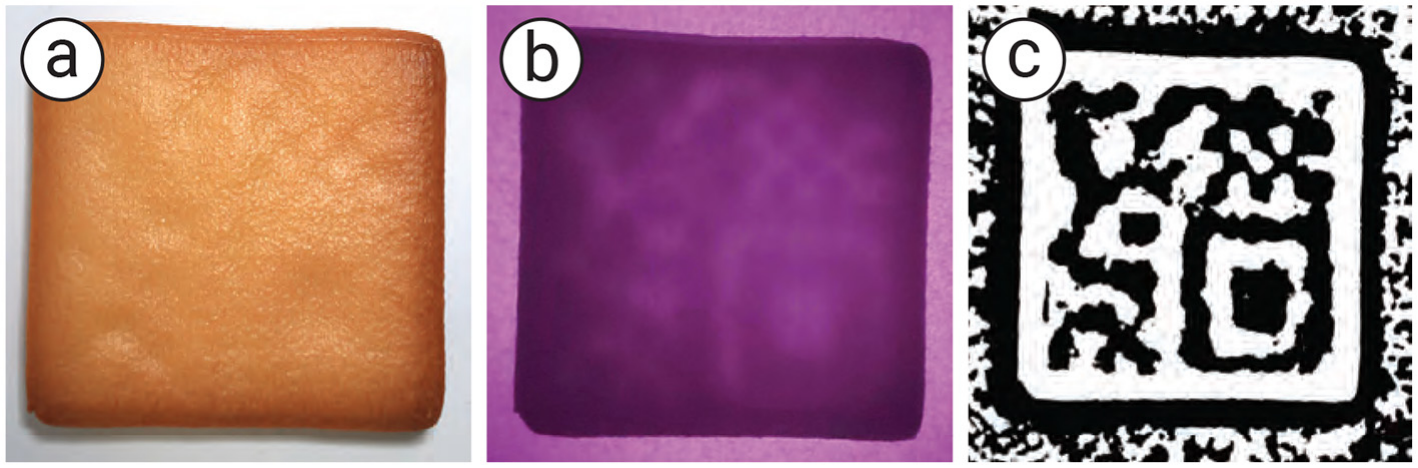
Back illumination with infrared light: **(a)** the user's naked-eye view, **(b)** the food captured under infrared camera, and **(c)** the image processing to recognize the tag.

Although the purpose of this experiment was to investigate the transmission spectra of backlight illumination in the visible wavelength range, we also conducted tests using an invisible wavelength at 880 nm (infrared light). As shown in Figure 9, the embedded pattern of the food tag containing air space, when illuminated with infrared light (Advanced Illumination Backlight, 880 nm), was successfully captured by an infrared camera (MQ013CG-ON, Ximea camera with a visible-cut/infrared-pass filter HWB800) and recognized using the same image processing software.

However, the infrared light did not transmit effectively through black cookie dough due to its absorption in the infrared spectrum. We plan to further investigate absorption spectra that differ in the infrared range, for example by considering food components such as proteins, glucose, sucrose, and water (26). Incorporating such components as infill materials could potentially enable the creation of infrared-readable tags.

##### 2.6.1.2 Separation of transmissive and scattered light

The captured image of the tag embedded inside the cookie contains both transmitted and scattered light. While the transmitted light passes directly through the infill structure, the scattered light degrades the image quality and reduces the tags readability. In particular, food tags printed without air space tend to contain more scattered light, further diminishing readability.

In this experiment, we extracted the transmitted light by applying a decomposition method based on high-frequency illumination (27) and measured the resulting tag readability.

**Figure 10:**
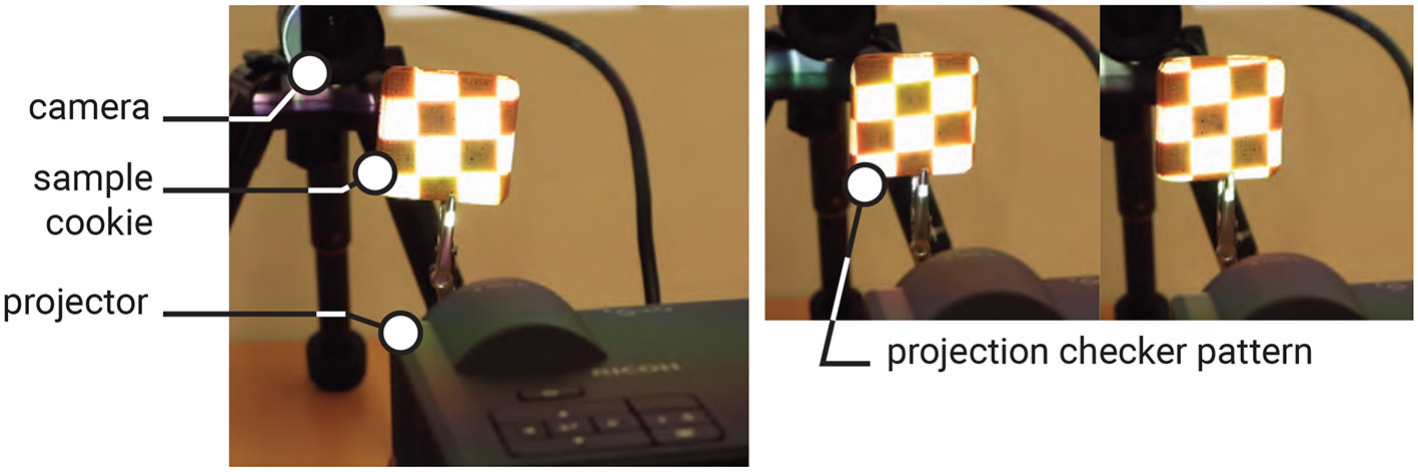
Checkered illumination setup to separate the transmissive and scattered lights **(left)**, and the sample of projection image **(right)**.

We replaced the white light illumination with a checkered pattern projection, shifting its phase multiple times during the image capture process (Figure 10). At each phase, we captured an image of the cookie and computed the maximum *L*_max_ and minimum *L*_min_ values of each pixel across the captured images. The decomposed transmitted and scattered light components are given by:


(1) $$\begin{array}{l} L_{t}\left[c\right]=L_{\text{max}}\left[c\right]-L_{\text{min}}\left[c\right] \end{array}$$

(2) $$\begin{array}{l} L_{s}\left[c\right]=2L_{\text{min}}\left[c\right] \end{array}$$

where *L*_*t*_ denotes the transmitted light, *L*_*s*_ denotes the scattered light, and *c* denotes a pixel index.

**Figure 11:**
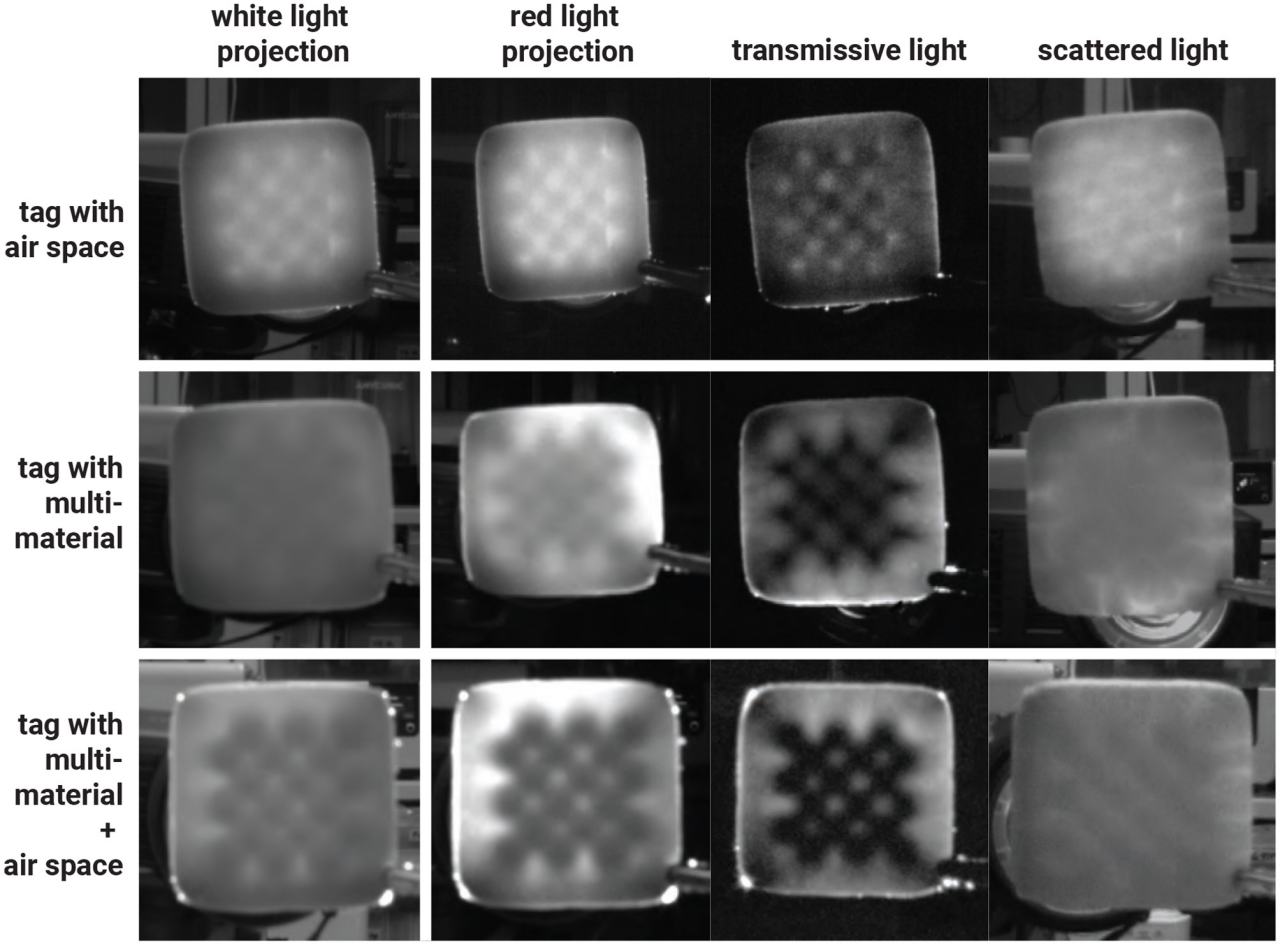
Results of tags captured under transmissive and scattered light separation method with various infills.

The results are shown in Figure 11. We repeated the procedure from the previous experiment, capturing images of the tags from different distances. Overall, tags were more easily recognized by our software when scattered light was removed.

Separating the scattered light from the transmitted light improved the maximum recognition distance from 21 cm to 23 cm for air-space tags, and from 24 cm to 29 cm for black cookie dough tags. In addition to air space and multi-material printing, we combined both approaches by printing tags using multiple materials with a 1 mm air gap. With this method, the tag could be recognized from as far as 35 cm using our software.

#### 2.6.2 Tag concealability

**Figure 12:**
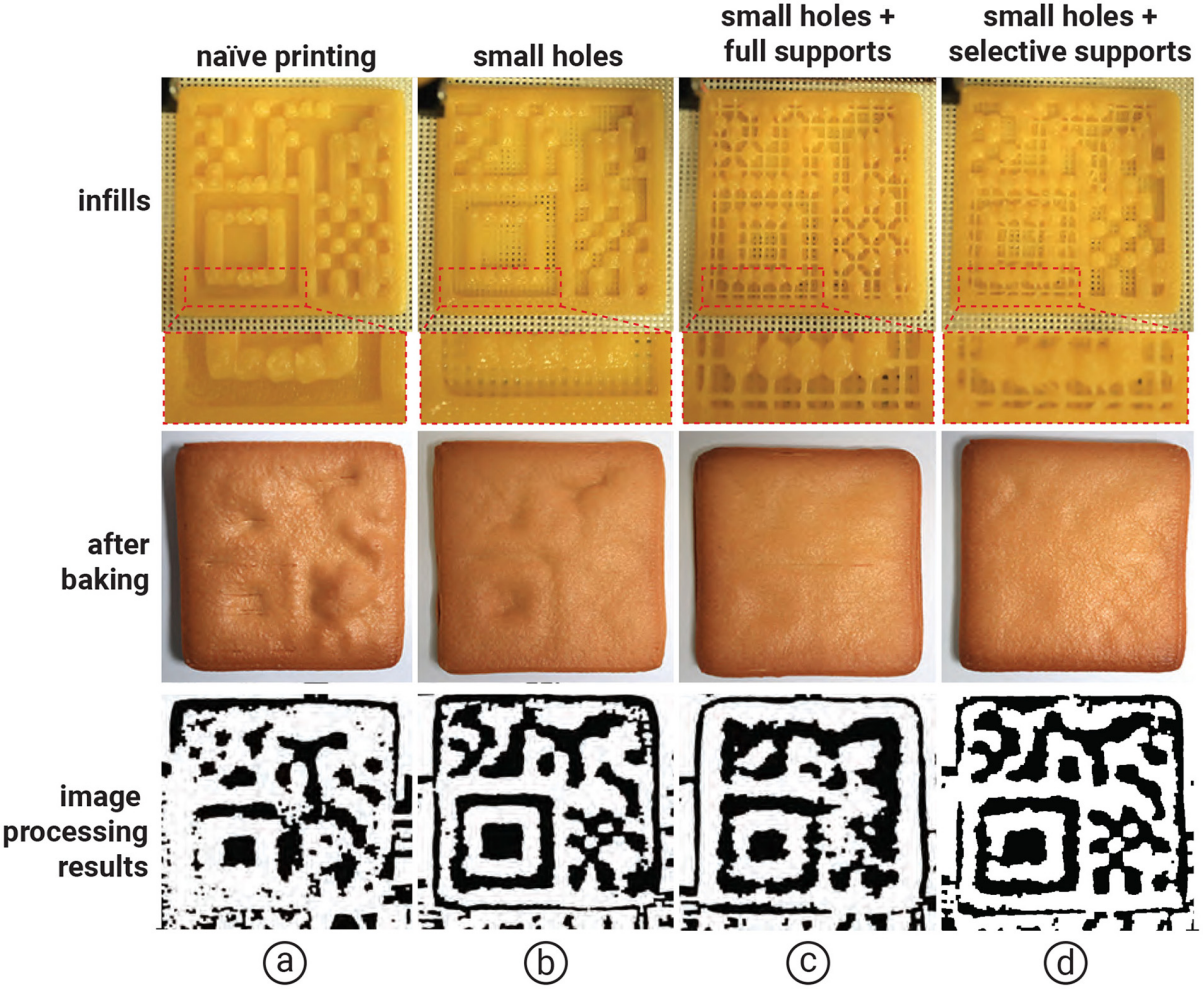
Results of adding the selective printing of supports over the hanging surface compared with **(a)** naïve, **(b)** small holes at the bottom of cookie, and **(c)** small holes with full support, respectively. Internal structures were inspected using a visible backlight and an RGB camera **(d)** small holes with selective supports.

Although our aim is to embed the tag inside the food so that it is less visible to users, one of our methods that uses air spaces inside the food can make the embedded tag visible after baking due to the expansion of air. The surface above the air pockets rises, making the shape of the tag visible (Figure 12a). In addition, printing cookie dough over the air space causes the dough to sag, also making the tag shape visible after baking.

Although we can reduce air expansion by 3D printing the cookie on a mesh baking sheet and adding *small holes* at the bottom of the cookie (Figure 12b), some areas still rise or sag during baking. To address this issue, we propose creating small supports over the air spaces to prevent rising and sagging during baking. For each air pocket, we print a 0.6 mm line of cookie dough to support the overhanging surface.

We conducted an experiment comparing selectively created supports over large air spaces (>16 mm^2^), full supports (i.e., supports over all air spaces), and no supports (i.e., naïve). As shown in Figure 12, we found that selectively creating supports over air spaces larger than 16 mm^2^ prevented the dough from rising or sagging and maintained the tags readability in the software, compared with full supports, which take more time to print, and small holes, which reveal the shape of the tag.

#### 2.6.3 Minimum tag size

Since our tagging method relies on the amount of infill, it is possible that the tag will not cover the entire cookie but instead be attached to only part of it. Therefore, it is important to evaluate the capability of our method to produce tags of different sizes. In our case, the standard size of a tag that is 100% readable at a distance of 15 cm is 4 mm per module, which corresponds to 52 mm × 52 mm for a micro QR code (13 × 13 modules) and 24 mm × 24 mm for an ArUco marker (6 × 6 modules).

We reduced the module size to 3 mm and 2 mm, resulting in overall tag sizes of 39 mm × 39 mm and 26 mm × 26 mm for the micro QR code, and 18 mm × 18 mm and 12 mm × 12 mm for the ArUco marker, respectively. While the 3 mm-module tag is readable from the same distance as the 4 mm-module tag, tags printed with 2 mm modules require capturing the image at a distance of 8 cm. Moreover, at 2 mm, the tag does not adhere well to the shells during printing, making fabrication more difficult. Therefore, we conclude that our tags are both printable and readable when the module size is at least 3 mm, using a 0.6 mm nozzle.

For thickness, we tested the tag at various values. While the tag can be produced with a thickness between 2 mm and 7 mm, we found that a thickness of 7 mm makes the readability unstable, whereas a thickness of 2 mm makes the printability unstable. Our results indicate that a thickness of 5 mm is optimal, as it avoids both readability and printability issues.

#### 2.6.4 Safety and eating experience

One of our goals is to print an edible tag that users can safely consume while enjoying the eating experience. We describe our setup in terms of the safety factors of our fabrication process and conduct experiments to verify whether users can enjoy the eating experience.

For food safety, our fabrication pipeline uses a one-time, food-dedicated syringe and an oil-free air compressor (California Air Tools 10020C) as 3D printer components to minimize bacterial contamination and other artifacts during the printing process. In addition, we conduct the fabrication experiments in compliance with the regulations of our local university.

For the eating experience, we conducted a pilot study with nine participants (aged 21–35) recruited from a local university. Participants were asked to eat 3D-printed cookies with an embedded tag created using (1) infill and air space, and (2) multi-materials (e.g., tags made from black food coloring), and to compare them with a cookie printed with 100% infill as the baseline. The participants were unaware of the infill structure of each cookie, as the cookies appeared similar from the outside.

They were asked to rate the mouthfeel (see (28) for details) in terms of dryness, hardness, smoothness, suppleness, and sweetness for each of the three cookies, using a 7-point Likert scale, to assess the similarity of the eating experience. The experimental protocol was approved by the Institutional Review Board (IRB) of the local university.

**Figure 13:**
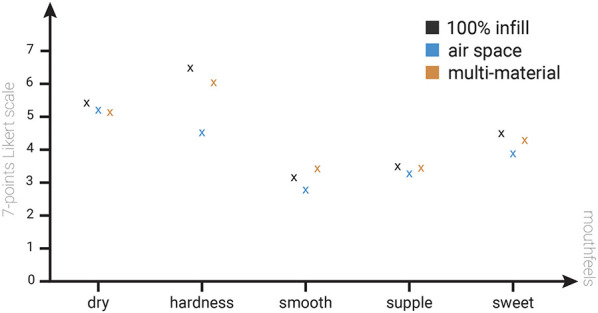
Results of eating experience experiment comparing two different tag embedding methods with 100% infill cookies.

Overall, participants reported that the three cookie types provided a similar eating experience. As shown in Figure 13, the perceived dryness was similar (avg. 5.5, 5.4, and 5.1 for 100% infill, air space, and multi-material, respectively), as were smoothness (avg. 3.1, 2.8, and 3.3), suppleness (avg. 3.3, 3.2, and 3.25), and sweetness (avg. 4.3, 4.0, and 4.1). However, participants perceived differences in hardness: the air space cookie averaged 4.35, and the multi-material cookie averaged 6.1, compared to the 100% infill cookies 6.5. These differences are attributed to the variation in infill structures.

## 3 Molding and stamping methods

**Figure 14:**
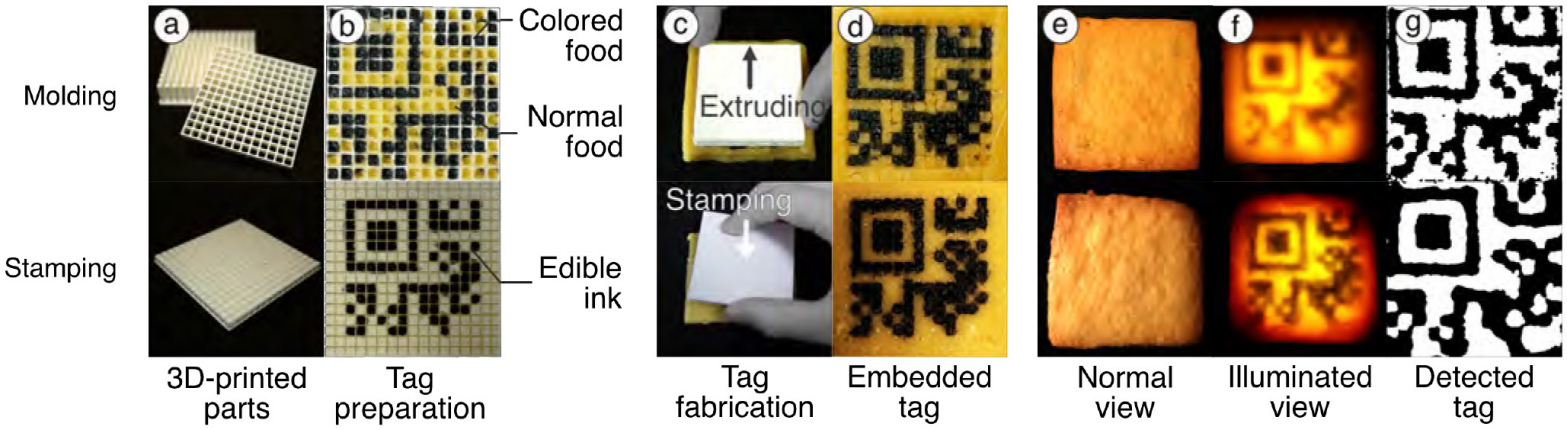
The overview of the proposed methods. **(a, b)** The 3D-printed mold and stamp prepared for tag embedding, **(c, d)** the embedding step, **(e, f)** tag visibility in normal and illuminated view, and **(g)** extracted tags via image processing.

While the food 3D printer method allows for precise tag generation, it is limited by the availability of compatible food materials and the fact that food 3D printers are not commonly found in household kitchens. To overcome these limitations and make the proposed method more accessible, we also developed alternative approaches using molding and stamping techniques inspired by traditional cooking practices. These techniques do not require specialized hardware (i.e., a food 3D printer) and rely only on conventional cooking skills, making them more accessible to chefs who lack technical expertise. Moreover, since these methods do not depend on nozzle-based extrusion, they allow the use of a broader range of food types, including those unsuitable for 3D printing. Figure 14 shows an overview of the proposed methods and sample outcomes. To evaluate the feasibility of these approaches, we conducted a small workshop in which home chefs created data-embedded foods using the molding and stamping techniques. We then assessed the resulting error rates.

### 3.1 Workflow of molding and stamping methods

The detailed workflow for embedding data using the molding and stamping methods is described in the following sections. For tag recognition, the same setup used in the 3D food printing system can also be applied to the molding and stamping methods, as described in.

#### 3.1.1 Molding method

Molding—filling a mold with material to form a desired shape—is commonly used in both the food industry and everyday cooking. It has also been employed in human-food interaction research to create computationally controlled food textures (6, 7, 24).

The molding method utilizes 3D-printed molds to discreetly embed edible tags into food. Each mold consists of two parts: a base with multiple cell-shaped cavities and an extruder with matching protrusions that fit into these cavities to form the tag pattern. These molds can be fabricated using a standard 3D printer. This method supports the data embedding strategies described in Sections 2.4.1, 2.4.2. For example, when using a multi-material strategy, the “0” regions of the tag are filled with a standard food material (e.g., cookie dough), while the “1” regions are filled with a contrasting material (e.g., blackened dough). The extruder is used to accurately position the tag structure within the mold, after which the tag is covered with a top layer to conceal it, as shown in Figures 14a–d Top.

Compared to the 3D printing method, the molding method can accommodate food materials that can be poured or pressed into molds. This makes it possible to use materials that are unsuitable for food 3D printers due to their hardness or large particle size. On the other hand, the molding method cannot employ the adaptive infill ratio control described in Section 2, as it lacks the ability to precisely control the amount of material filled into the mold. As a result, it can only use one of the data embedding strategies—either air gaps or multi-materials—but cannot combine both within a single tag.

#### 3.1.2 Stamping method

Drawing on food surfaces with edible ink is a common practice in everyday cooking, often used for intricate decorations such as stamping patterns onto icing cookies (29).

Inspired by this technique, we enabled data embedding by stamping edible ink onto the interior surface of food. We use a 3D-printed stamp equipped with a guide that defines the tag's cell layout. To embed a tag, the cells corresponding to the “1” bits are painted with black edible ink. The stamp is then pressed onto the target area, transferring the ink to the foods surface. Finally, the tagged area is covered with a top layer to conceal the tag from plain view, as shown in Figures 14a–d Bottom.

Compared to 3D printing, the stamping method enables tag creation in food materials where edible inks can be transferred from the stamp, regardless of the materials hardness or particle size. This allows the use of food types that are unsuitable for 3D printing. On the other hand, the stamping method cannot control the infill ratio of food materials, as it does not manipulate the internal structure of the food in its data embedding process.

### 3.2 Evaluations

#### 3.2.1 Tag detectability

To verify the embeddability and detectability of the molding and stamping methods, we created data-embedded samples using these methods. We printed a plastic mold and stamp using a standard 3D printer (Ultimaker Ultimaker S3) with 13 cells, each 3.6 mm in size, a total of 55.2 mm for each method. To make samples, we used cookie dough made of flour, sugar, egg yolk, and butter in a 1.0:0.5:0.2:0.5 ratio, which is empirically unsuitable for 3D food printers due to its hardness. We embedded the data by molding and stamping methods into the dough rolled out to an even thickness of 5 mm. The dough with the data was covered with a 1 mm layer of another cookie dough to conceal it from normal view and baked in an oven for 20 min.

We confirmed that the tags emerge when the cookie is illuminated from the backside but remain invisible to the naked eye without illumination (Figures 14e–f). To check the detectability, we placed a camera on the top and a light on the bottom of the sample and experimented with simple image processing: denoising (Gaussian blur), contrast boosting (CLAHE), and binarization. We confirmed that these processed tags can be read by off-the-shelf tools such as QRQR (Denso Wave, Figure 14g). The result found that the embedded tags with the proposed methods can be detected through commercially available tools.

#### 3.2.2 Tag concealability

**Figure 15:**
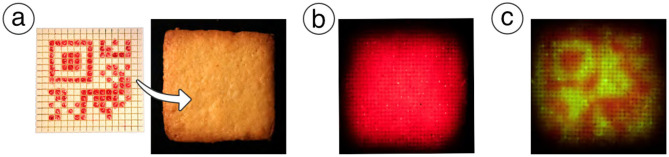
Use of other colors for tags. **(a)** Tag embedding using edible red ink. The tag is invisible in normal view. **(b)** The tag is still invisible under red illumination. **(c)** The tag becomes visible under green illumination.

We confirmed that the 1 mm of the top layer is enough to conceal the tag from the naked eye in normal lighting conditions. On the other hand, we also found that embedded tags become slightly visible to the naked eye even without light illumination when the cover layer is too thin. To overcome the visibility of the tag, we additionally explored appropriate color combinations for the tag and illumination to reduce surface contrast by the tag in normal view while retaining detectability during illumination. We confirmed that the visibility of tags to the naked eye can be reduced while still being detectable when using a red tag and green illuminations for cookies (Figure 15).

### 3.3 Mini-workshop: stability of data embedding and extraction

We conducted a mini workshop to evaluate whether non-technical creators could fabricate data-embedded food using our proposed methods and whether the embedded data could be reliably extracted.

#### 3.3.1 Setup for the workshop

Three home chefs participated in the workshop and were asked to create three cookies each, using both stamping and molding techniques. At the beginning, we provided a live demonstration of the fabrication methods. Following this, participants proceeded at their own pace, while the facilitator handled preprocessing (e.g., material preparation and dough making) and postprocessing (e.g., baking and cooling) to maintain consistency.

**Figure 16:**
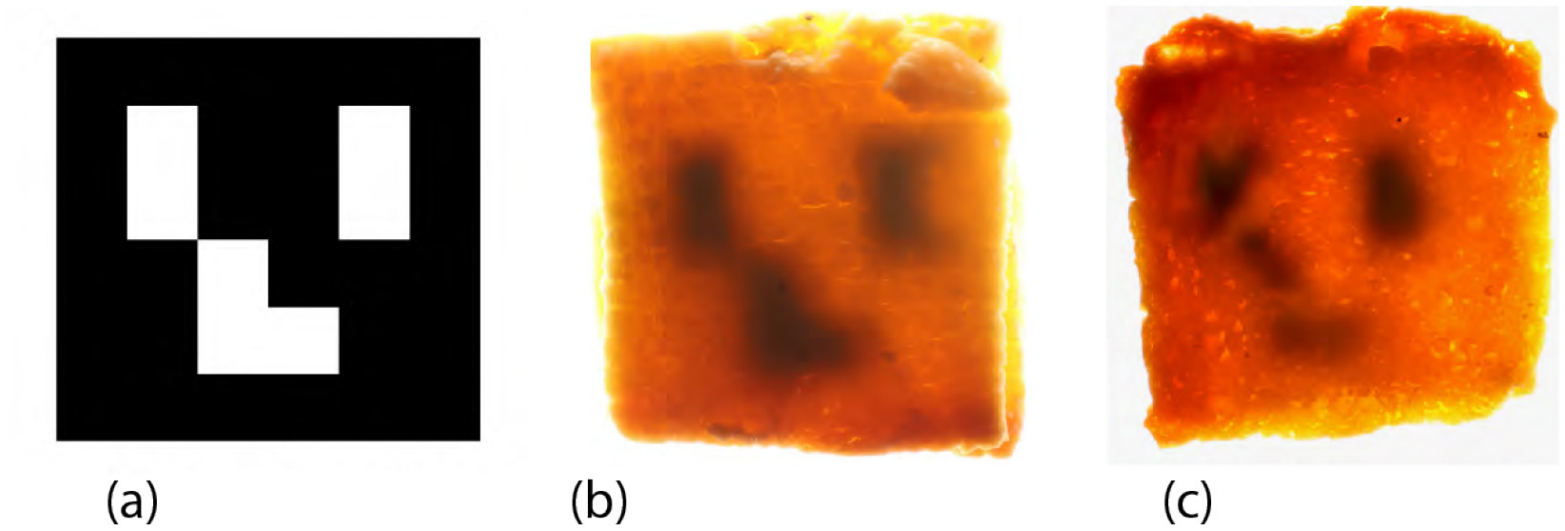
Results of data embedding in the workshop. **(a)** The target tag is used for embedding. **(b)** A successful sample under backlight illumination, in which the tag structure was preserved after baking. **(c)** A failed sample under backlight illumination, where the tag became unrecognizable, likely due to structural collapse and mixture.

We used cookie dough as the base material, layered with black-colored dough prepared according to the procedure described in Section 3.2.1. An ArUco marker was selected as the target tag (Figure 16a).

#### 3.3.2 Result of data extraction

**Figure 17:**
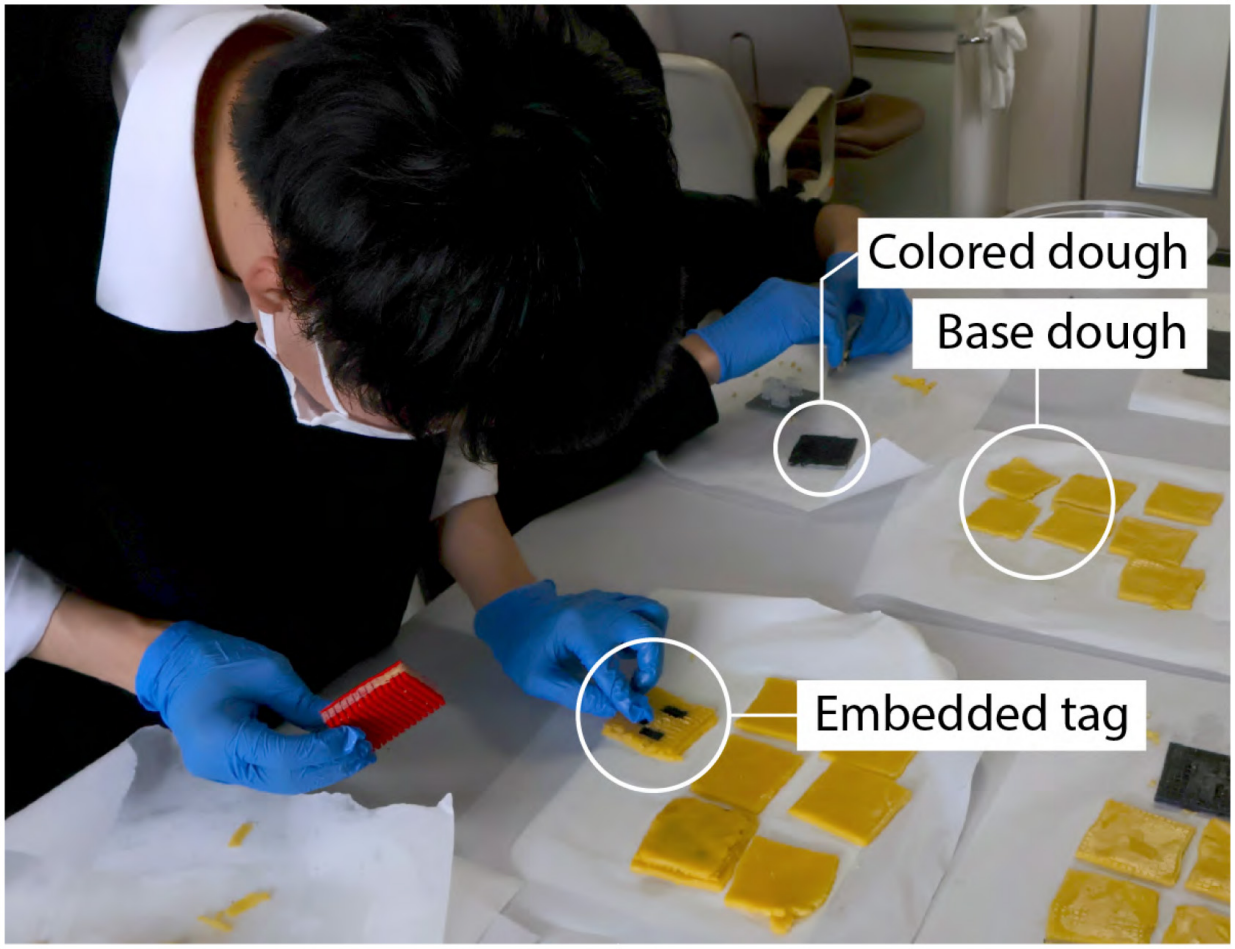
A captured scene during the workshop.

Figure 17 presents a captured scene from the workshop. Table 1 summarizes the extraction results. Overall, approximately 67% of the tags were successfully recognized using the image processing pipeline described in Section 2.5. Notably, all the samples produced by Participant A using both the stamping and molding methods were correctly recognized. An example of a successful sample is shown in Figure 16b, where the tag structure remained intact after baking.

**Table 1:** Data extraction results for three samples from the workshop.

	**(a) Molding method**				**(b) Stamping method**		
	**Sample 1**	**Sample 2**	**Sample 3**		**Sample 1**	**Sample 2**	**Sample 3**
Participant A	✓	✓	✓	Participant A	✓	✓	✓
Participant B	Failed	Failed	✓	Participant B	Failed	Failed	✓
Participant C	Failed	✓	✓	Participant C	Failed	✓	✓

Outcomes are shown for each participant when using (a) the molding method and **(b)** the stamping method (✓: successful extraction, Failed: unsuccessful). The results suggest variability in success rates depending on both the method and participant.

However, we also observed several failures, particularly in the early attempts by Participants B and C. As illustrated in Figure 16c, these failed samples exhibited deformed or indistinct tag patterns. Participant B's samples often had distorted or blurred cells, likely due to unintentional deformation during shaping. Participant C's first attempt failed because the normal and colored doughs were excessively mixed, making the pattern unrecognizable. Despite these initial issues, participants reported increased confidence and improved outcomes after creating one or two samples. This learning effect was reflected in the results: all final samples successfully yielded extractable tags. Although our workshop involved only three participants, the consistent improvement after initial trials suggests that basic fabrication skills can be acquired with minimal instruction. Future work will evaluate scalability with a larger user base.

To summarize the distinctions among the three fabrication methods, we provide a comparative overview in Table 2. The table outlines key differences in terms of material compatibility, ease of use, tag capacity, advantages, and limitations. This comparison highlights the trade-offs between technical precision and accessibility, offering guidance for selecting an appropriate method based on the target cooking context and user expertise.

**Table 2:** Comparative summary of the three embedding methods.

**Method**	**Material compatibility**	**Ease of use**	**Tag capacity**	**Pros**	**Cons**
3D printing	Limited to paste-like materials	Requires technical setup and 3D printer	High (supports complex geometries)	High precision; customizable geometry	Time-consuming; requires specialized equipment
Molding	Supports moldable materials	Moderate; uses standard tools and molds	Medium	Accessible; reproducible without a printer	Limited shape flexibility
Stamping	Suitable for food surfaces that allow edible ink transfer	Easy	Medium	Fast; stamp is reusable	Ink diffusion possible

## 4 Discussion and limitations

We discuss limitations and future work that could potentially improve the tagging of food data using 3D printing, molding, and stamping methods.

### 4.1 Applicability beyond cookie dough:

**Figure 18:**
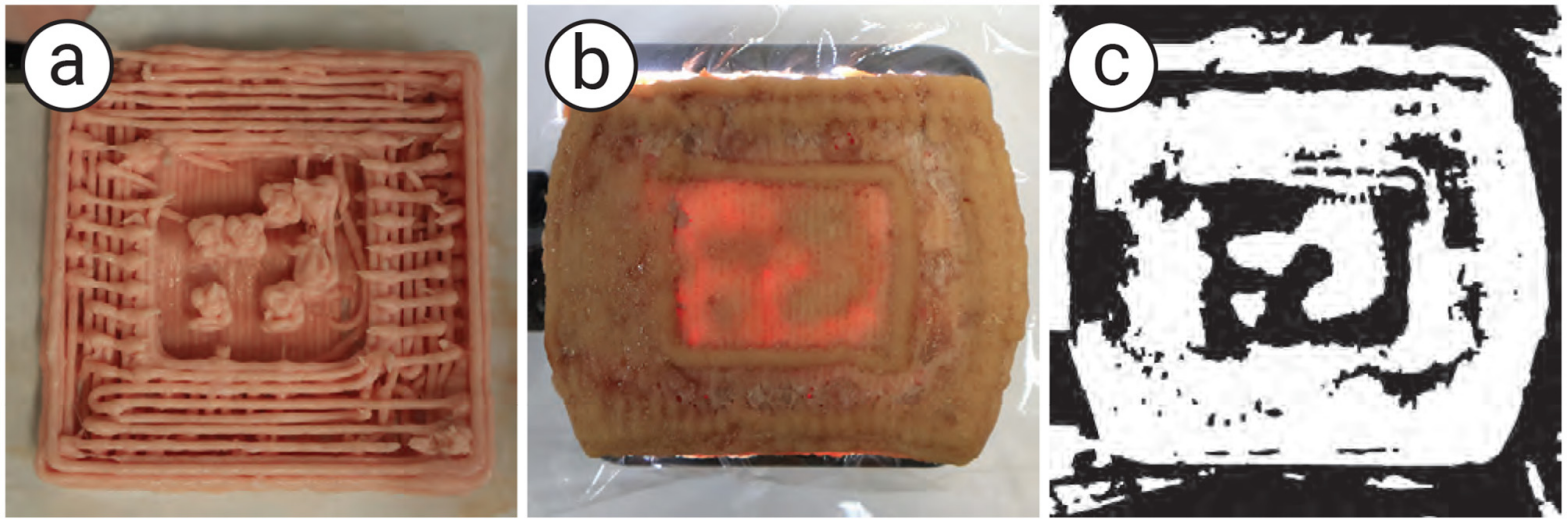
3D printed meat with a tag. **(a)** The outlook during the printing process, **(b)** the meat under back illumination, and **(c)** the image processing result.

In our 3D printing method, we primarily focused on cookie dough as the base material, but the technique can also be adapted to other extrudable materials such as ground meat, as shown in Figure 18. However, materials that are not suitable for extrusion cannot be used with 3D printing alone. To address this limitation, we introduced molding and stamping techniques, which expand the range of compatible food materials beyond those usable in 3D printers. Nevertheless, our current pipeline still requires that the material be fillable into molds or transferable via stamps. We believe that incorporating laser cutters (11, 23, 30) to cut patterns or brown surfaces could further extend data embedding to a broader range of food materials. As future work, we plan to explore this direction while carefully evaluating safety aspects, drawing on prior studies that demonstrated controlled laser use for food browning and surface decoration without adverse effects on common ingredients.

### 4.2 Information capacity

In the 3D food printing method, increasing the tag size to embed more information is limited by the longer printing times. The extended process can cause the outer shell to collapse due to changes in the material properties during printing. In contrast, the molding and stamping methods can embed tags much faster, as they do not rely on a layer-by-layer fabrication process. Therefore, these methods can overcome the limitation of embedding larger tags.

Currently, our minimum readable module size is 3 mm square. Embedding a version 1 edible QR code therefore requires at least 63 mm square (3 mm × 21 modules). This requirement is consistent across all methods, as it depends primarily on the optical transmission properties of the food materials. To further reduce the module size, we need to explore food materials that exhibit more distinct spectral transmission contrasts between the tag and its surroundings. For example, using transparent jelly as a base and embedding quinine, which fluoresces under ultraviolet light, could allow for smaller and more compact tags, since transparent materials transmit more light than opaque ones.

### 4.3 Tag concealability vs. visibility

Our technique conceals tags by adding a top layer above the tag layer. A thickness of 1 mm is sufficient to hide tags in cookies under normal illumination, though thicker layers improve concealment at the cost of backlight visibility due to scattering. To mitigate this trade-off, we applied phase-shifted projection to remove scattered light, enabling thicker layers without visibility loss. We also used color matching to reduce surface contrast, allowing thinner layers for improved visibility. Future work will combine these approaches for broader material compatibility.

### 4.4 Visibility of back illumination

Our technique supports backlight illumination in both visible and invisible spectra, but currently achieves its best performance with food tags containing internal air spaces. In contrast, multi-material printing typically requires visible-spectrum illumination for recognition, which can make the tag faintly visible to users during operation. To address this issue, we plan to explore material combinations with transmission properties tuned to invisible wavelengths, such as infrared. Additionally, implementing high-speed synchronization between illumination and camera capture could enable momentary, imperceptible exposure of the tag (31). As a more immediate workaround, we also consider embedding visually natural alternatives, such as text or binary patterns, to further enhance unobtrusiveness.

### 4.5 Number of participants

We employed nine participants in the eating experience study, i.e., to understand the effects of infills and the taste of the food sample. Although we did not establish specific evaluation criteria, we compared the eating experiences using the Just Noticeable Difference (JND) method. Given that the current sample size is relatively small, our main contributions rely on the fabrication methods to embed data as the edible tag. We plan to increase the number of participants in future studies and conduct a more rigorous statistical evaluation.

In addition, we conducted a preliminary workshop with three home chefs to demonstrate the applicability of the molding and stamping methods for non-expert users. We intend to expand the participant pool to include a broader range of individuals with varying levels of culinary expertise. This expansion will enable us to more systematically evaluate the accessibility and robustness of our methods.

## 5 Conclusion

We introduced a data-embedding pipeline for the 3D printing approach and extended its applicability to a wider range of food materials through molding and stamping. We evaluated these methods in terms of tag detectability, concealability, material compatibility, and stability of data extraction. The results demonstrated that the methods are applicable to diverse food types (e.g., 3D-printable, moldable, and stampable foods) and that the embedded tags can be detected using off-the-shelf tools. We also conducted a workshop with home chefs to investigate the accessibility of the molding and stamping methods, and found that they could successfully create the tags without prior training. Finally, we discussed limitations and outlined future directions for improving food data tagging.

## Data Availability

The datasets presented in this article are not readily available because the dataset is not shared in this work. Requests to access the datasets should be directed to miyatake.yamato@gmail.com.
